# A85 ASSESSING THE IMPACT OF A DEDICATED RAPID INTERDISCIPLINARY IBD CLINIC ON PATIENT WAIT TIMES, REPORTED OUTCOMES, AND SATISFACTION OF CARE

**DOI:** 10.1093/jcag/gwac036.085

**Published:** 2023-03-07

**Authors:** G Mathiyalagan, E Broni, R Jogendran, K O'Connor, E Kennedy, A Steiman, C Maxwell, A Omar, V Piguet, A Alavi, A Weizman, V Huang

**Affiliations:** 1 Mount Sinai Hospital; 2 Women's College Hospital, Toronto, Canada

## Abstract

**Background:**

Inflammatory bowel disease (IBD) is a chronic fluctuating condition where patients can experience periods of active disease and remission. Timely access to care has been shown to be impactful on important disease outcomes. In January 2020, we implemented a rapid assessment IBD program consisting of expedited access to interdisciplinary care and close monitoring of patients.

**Purpose:**

To assess the impact of the rapid assessment program on access to care, disease activity, and patient satisfaction.

**Method:**

Once informed consent was obtained, patients were enrolled into the RAPID IBD program. This program consisted of four close monitoring time points at baseline, 1, 2, and 3 months, as well as two follow up time points at 6 and 12 months. At each timepoint patients completed questionnaires that evaluated disease activity, using the Modified Harvey Bradshaw Index (mHBI) for Crohn’s disease (CD), partial Mayo (pMayo) score and Simple Clinical Colitis Activity Index (SCCAI) for ulcerative colitis patients. At baseline, 3, 6, and 12 months, patients were also assessed on mental health, using the Patient Health Questionnaire (PHQ-9) and General Anxiety Disorder (GAD-7), and satisfaction of care, using the CACHE questionnaire.

**Result(s):**

Between January 2020 – August 2021, 216 patients were referred to the RAPID IBD program. The mean time from referral to clinical assessment was 8.1 days. Of those referred, 143 (71 CD, 62 UC, 6 IBDU, and 4 Query IBD) patients consented to and completed the 12-month RAPID IBD study. 34.9% of patients who had active disease at baseline achieved remission by 3 months (Table 1). At baseline 44.8% and 28.4% of patients experienced moderate to severe depression and anxiety, respectively. The greatest improvement in mental health was seen at 2 months where the proportion of patients experiencing moderate to severe depression and anxiety decreased to 27.5% and 18.3%, respectively (Table 2). Patient satisfaction, specific to clinical care, improved from a baseline score of 69.1% to 74.1% at 3 months (Table 3).

**Image:**

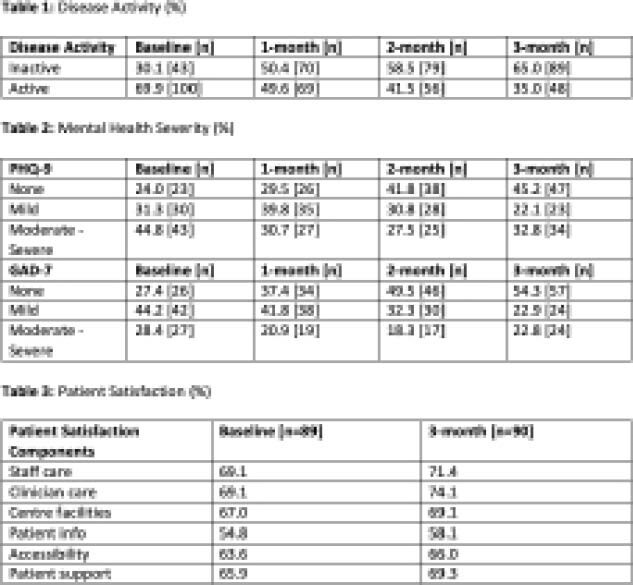

**Conclusion(s):**

Implementation of a dedicated RAPID IBD clinic program reveals shorter wait times to be seen in clinic. By three months of enrollment, patients demonstrate improvements in clinical response, mental health, and satisfaction of care.

**Please acknowledge all funding agencies by checking the applicable boxes below:**

Other

**Please indicate your source of funding;:**

AMO Innovation Funding

**Disclosure of Interest:**

None Declared

